# Treatment of localized gastric and gastroesophageal adenocarcinoma: the role of accurate staging and preoperative therapy

**DOI:** 10.1186/s13045-017-0517-9

**Published:** 2017-08-15

**Authors:** Brian Badgwell, Prajnan Das, Jaffer Ajani

**Affiliations:** 1 Department of Surgical Oncology, The University of Texas, Unit 1484, MD Anderson Cancer Center, 1515 Holcombe Blvd., Houston, TX 77030 USA; 2 Departments of Radiation Oncology, The University of Texas MD Anderson Cancer Center, Houston, TX USA; 3 Departments of Gastrointestinal Medical Oncology, The University of Texas MD Anderson Cancer Center, Houston, TX USA

**Keywords:** Gastric cancer, Preoperative treatment, Neoadjuvant, Chemotherapy, Chemoradiotherapy, Surgery

## Abstract

Gastric cancer is the third most common cause of cancer death worldwide, although it is not in the top 10 causes of cancer death in Northern America. Due to clear differences in incidence, screening, risk factors, tumor biology, and treatment between gastric cancers from Eastern and Western countries, our treatment is primarily guided by trials from Western countries. Patients undergo an extensive staging evaluation including high-quality CT imaging, endoscopic ultrasound, and diagnostic laparoscopy with peritoneal washings for cytology. Patients are presented in multidisciplinary conference with input from medical, radiation, and surgical oncology, in addition to further evaluation of existing studies and biopsy results by diagnostic radiology and pathology colleagues. Due to the well-documented difficulty in tolerating postoperative therapy, patients are frequently treated with preoperative chemotherapy and chemoradiotherapy. Extended lymph node (D2) dissection is routinely performed during subtotal or total gastrectomy. Ongoing trials in Western populations comparing preoperative chemotherapy to chemoradiotherapy will help inform the decision regarding the optimal treatment for patients with resectable gastric cancer. Additional studies are needed to identify predictors of treatment response to identify the optimal preoperative or perioperative approach. As peritoneal disease is the most common site of recurrence, studies are also urgently needed for more accurate methods of detecting peritoneal disease at diagnosis, and also investigating potential treatment modalities such as hyperthermic intraperitoneal chemotherapy.

## Background

Gastric cancer is a leading cause of cancer death worldwide, although only the 15th most common cause of cancer death in the USA [1, 2]. Geographic variations in incidence rates are likely multifactorial and may be due to differences in diet, food preservation, familial risk, and *Helicobacter pylori* infection rates. The biology of gastric cancer in Eastern Asia may be very different from the biology of tumors found in Northern America and Europe which demonstrate higher rates of poorly differentiated histology, signet ring cell histology, and proximal stomach involvement—variables which are all associated with poorer survival. The high incidence rates of gastric cancer in Eastern Asian countries has also led to the adoption of screening upper endoscopy, which results in the identification of asymptomatic, early stage gastric cancers. The differences in incidence, screening, risk factors, tumor biology, and treatment result in a wide gap in survival outcomes between Eastern and Western countries that limits the generalizability of studies between regions, as patients from Eastern Asia demonstrate markedly improved survival. Table 1 demonstrates the variation in 5-year overall survival rates between randomized clinical trials of surgery and additional therapy from Eastern and Western regions. Treatment in Eastern countries is also notable for the preference for a surgery first approach as many early cancers do not require adjuvant therapy, and radiation therapy is infrequently considered. Conversely, all of the Western trials in Table 1 utilize some component of preoperative therapy, making cross trial comparison of studies from Eastern and Western regions, based on pathologic stage, quite difficult. As current U.S. national guidelines pertaining to locoregional gastric cancer are based primarily on Western studies, with the exception of adjuvant chemotherapy, we will focus primarily on treatment trials and recommendations from North America and Western Europe that guide our current practice.

**Table 1:** Five-year overall survival rates for randomized clinical trials of surgery and additional therapy, stratified by trial location (Eastern vs. Western countries)

Trial	Surgery Only	Surgery + Chemotherapy	Surgery + Chemoradiotherapy
Eastern			
ARTIST [39]		73%	75%
ACTS-GC [40]	61%	72%	
Western			
CRITICS [41]		41%	41%
CROSS [16]	34%		47%
MAGIC [42]	23%	36%	
FNCLCC/FFCD [43]	24%	38%	

The lifetime risk of gastric cancer in the USA is approximately 1%, with a 5-year survival rate of 30% for all stages based on recent Surveillance, Epidemiology, and End Results registry data [2]. Five-year relative survival rates for patients with localized, regional, and distant disease are 67, 31, and 5%, respectfully, highlighting the limitations in our current treatment for advanced disease and the benefit to earlier detection of resectable disease [2]. The purpose of this review is to summarize the recent developments in the treatment of locoregional gastric and gastroesophageal adenocarcinoma. Although there are some ongoing minor controversies regarding the extent of surgery, much of the recent improvement in the treatment of gastric cancer is attributed to the addition of chemotherapy or chemoradiotherapy. Due to our institutional preference for preoperative therapy, we will also summarize our multidisciplinary approach to staging and treatment for patients with gastric cancer.

## Multidisciplinary conference

New patients arriving at our center are presented in a weekly multidisciplinary conference after initial staging with routine attendance by medical oncology, radiation oncology, surgical oncology, pathology, and radiology. Attendance by advanced practice providers as well as basic science researchers also facilitates more streamlined clinical care and integration of research projects. Additional medical professionals not in attendance, but in close communication, include gastroenterologists, thoracic surgeons, and geneticists. Although we have not published data on the frequency of the change in management based on multidisciplinary review, anecdotally we identify previously undescribed radiologic findings on the order of 10–15%. Although awaiting presentation in the conference may delay the initiation of therapy by approximately a week, the scheduling of the endoscopic ultrasound and laparoscopy with port placement are ongoing during this time.

## Preoperative staging

Clinical staging is critical to treatment, as outlined in current national guidelines, but also as highlighted in the most recent American Joint Commission on Cancer TNM Staging system. The clinical stage in the 8th edition of TNM staging is defined prior to treatment based on endoscopy (possibly including endoscopic ultrasound with fine needle aspiration), imaging, and diagnostic laparoscopy with washings [3]. However, there are several limitations in the accuracy of radiologic, endoscopic, and laparoscopic staging. Endoscopic ultrasound has acceptable accuracy in distinguishing T1 from T2–T4 lesions, which is important for deciding whether to administer preoperative therapy. However, both CT imaging and endoscopic ultrasound have low sensitivity for determining nodal status which is not as critical of a limitation when T2 or greater patients are treated preoperatively and T1 patients have overall low rates of nodal involvement [4].

Staging including endoscopic ultrasound can identify the rare patient in the USA that presents with an early stage gastric cancer suitable for consideration for endoscopic mucosal resection or endoscopic submucosal dissection. Current National Comprehensive Cancer Network Guidelines suggest this treatment is adequate for patients with a lesion ≤2 cm, well or moderately well differentiated histology, that does not penetrate beyond the superficial submucosa (early T1b), does not exhibit lymphovascular invasion, and has clear lateral and deep margins [5]. Few tumors in Western populations meet this rigorous criteria, and the skills and instrumentation required for endoscopic submucosal dissection are not widely available in the USA.

Laparoscopy with peritoneal washings for cytology is a high-yield, low-risk routine aspect of preoperative staging. In our patient population in which 84% had T3 tumors and 66% were node positive on endoscopy, laparoscopy identified carcinomatosis in 21% and positive cytology only in another 13%. With the addition of a few other important findings, such as liver cirrhosis or locally invasive tumors, the overall yield with laparoscopy was 36% [6]. Performing peritoneal washings is of critical importance in staging, as positive peritoneal cytology represents stage IV disease according to the American Joint Commission on Cancer Staging system [3]. Current National Comprehensive Cancer Network guidelines also note that carcinomas with positive cytology are considered unresectable with treatment recommendations of systemic therapy or best supportive care [5]. The practice of initiating chemotherapy without laparoscopy, in potentially resectable patients, with the plan to perform laparoscopy at attempted resection after preoperative chemotherapy is not advisable for a few reasons. First, chemotherapy can convert positive cytology to negative and therefore submit a patient to resection in the setting of a history of stage IV disease. Second, cytology from peritoneal washings is best performed in a routine fashion with the availability of immunohistochemical stains and collaborative assessment as immediate intraoperative analysis may not be as accurate. Third, a considerable delay occurs after stopping chemotherapy, waiting 4 weeks, performing the laparoscopy with attempted resection, identifying metastatic disease and aborting the resection, and then resuming chemotherapy. Although a patient in this scenario was spared the morbidity of an unnecessary laparotomy, the delay in continuing chemotherapy could be prevented with an upfront laparoscopy. Lastly, the identification of peritoneal disease at diagnosis allows for multidisciplinary planning regarding potential clinical trials.

## Preoperative, perioperative, or postoperative treatment

**Fig. 1:**
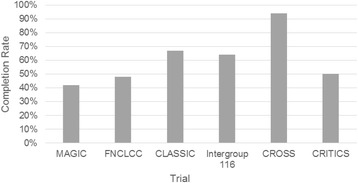
Completion rates for randomized clinical trials of perioperative or postoperative chemotherapy or chemoradiotherapy for resectable gastric adenocarcinoma

Upfront surgery is unusual and primarily utilized for early gastric cancer, defined as T1aN0 and T1bN0 lesions. Not infrequently, the endoscopist will not be able to tell the difference between a T1b and T2 lesion, and in those situations, we treat as the less invasive tumor with upfront surgery, to avoid the potential for overtreatment. Otherwise, we rarely approach tumors with upfront surgery, due to the oft-reported difficulty in administering therapy after gastrectomy. Figure 1 reports the completion rates for trials of perioperative or postoperative chemotherapy or chemoradiotherapy. Often, experts will refer to T2N0 lesions as early lesions, but our institutional experience is that patients with T2 cancers have a long-term survival rate of only 66% and we therefore consider those patients for full multimodality treatment [7].

## The MD Anderson Algorithm for advanced, resectable gastric cancer

**Fig. 2:**
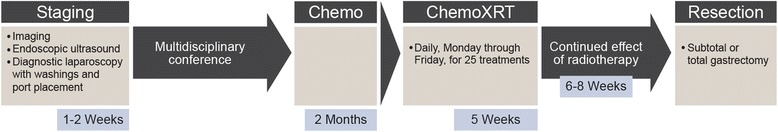
Algorithm and timeline for preoperative induction chemotherapy and chemoradiotherapy for patients with potentially resectable gastric adenocarcinoma. (Reprinted from MD Anderson Cancer Center, with permission)

After CT imaging, endoscopic ultrasound, and laparoscopy (with central venous catheter port placement), we initiate chemotherapy as soon as possible. Chemotherapy is most often 5FU and oxaliplatin administered every 2 weeks for 4 cycles (2 months total). Then patients undergo chemoradiotherapy of 45 Gy with concurrent 5FU/capecitabine, with or without oxaliplatin. Patients tend to experience the most severe side effects of chemoradiotherapy during the last week of treatment, and the first 2 to 3 weeks post treatment completion. We currently plan for surgery 7 to 8 weeks after completion of chemoradiotherapy, as the optimal time to allow for treatment response yet prior to tumor regrowth, but also to allow the patient to recover prior to gastrectomy. Figure 2 illustrates our general algorithm with timeline.

The choice of chemotherapy and length of treatment is an area of active debate. One study which guides our current practice is the OEO5 study, in which 2 cycles of cisplatin and fluorouracil was equivalent to 4 cycles of epirubicin, cisplatin, and fluorouracil or epirubicin, cisplatin, and capecitabine (ECF/ECX), based on overall survival and progression-free survival [8]. The mounting evidence suggests that epirubicin should not be used in patients with gastric adenocarcinoma [5, 9]. Although other investigators have supported the use of ECF/ECX or fluorouracil, oxaliplatin, and docetaxel, triplet regimens produce more toxic effects than doublet regimens, with 30-day mortality rates of 2–4% [10]. Further evidence against the use of taxanes comes from a randomized clinical trial showing modest survival advantage in the first-line advanced gastric adenocarcinoma setting [11]. Therefore, we most often treat patients with potentially resectable gastric cancer with 5FU and oxaliplatin, which also leaves more treatment options for the metastatic setting, should recurrence occur after surgery.

The use of preoperative chemoradiotherapy for gastric cancer is based on multiple phase II trials, which show a 20–30% pathological complete response rate from the combination of preoperative chemotherapy and chemoradiation [12–14]. In the postoperative setting, chemoradiotherapy results in a 9% improvement in overall survival compared to surgery alone [15]. Level 1 evidence also exits to support the use of preoperative chemoradiotherapy for tumors of the gastroesophageal junction [16]. The phase II trials on gastric cancer, in combination with trials from gastroesophageal junction cancers, have justified inclusion of preoperative chemoradiotherapy in current NCCN guidelines [5]. A large international randomized trial (TOPGEAR) is currently evaluating the role of preoperative chemoradiation for gastric cancer; interim results have shown that preoperative chemoradiation can be administered safely [17]. Although the investigators for this important trial utilized ECF for the chemotherapy regimen, they are planning to modify the trial to allow for the incorporation of docetaxel, oxaliplatin, and fluorouracil/leucovorin (FLOT)-type regimens based on recently presented results of a phase III trial demonstrating improved survival with FLOT versus ECF/ECX [18, 19]. The results of this trial will likely have a major impact on our current practice pattern and will influence national guidelines for the preoperative treatment of gastric cancer in Western populations, but the debate will continue regarding the optimal perioperative chemotherapy regimen. There are also some differences in the technique of chemoradiotherapy between the TOPGEAR trial and our institutional approach. The TOPGEAR trial investigators deliver radiotherapy to the entire stomach, any perigastric tumor extension, and regional lymph nodes. In brief, the technique at our institution varies slightly in that we utilize the results of our extensive preoperative staging including imaging, endoscopic ultrasound, and laparoscopy to map the primary tumor and treat with a minimum 4 cm mucosal margin along with involved nodes, as well as regional and D2 lymph node basins.

## Surgical resection

Resection is either subtotal or total gastrectomy with roux-en-y reconstruction. Non-anatomic (wedge) resection, such as in patients not candidates for formal gastrectomy, is rare and performed infrequently for palliation of symptoms of bleeding or local tumor control. Proximal gastrectomy is not currently performed due to concerns over the oncologic equivalency of this approach and severe reflux. Gastroesophageal junction tumors require a tailored approach based on the Siewert classification and the extent of gastric involvement. The outgoing AJCC guidelines classified gastric cardia tumors with up to 5 cm of gastric involvement as esophageal cancers, but thankfully this misclassification has been corrected in the 8th edition [20]. The 8th edition gastric cancer staging system now incorporates a post-neoadjuvant therapy classification, but unfortunately does not contain a complete pathologic response category, which can occur 18% of the time for patients treated with preoperative chemoradiotherapy [21]. Similar to esophageal cancer staging, patients with a pathologic complete response (ypT0N0) may be considered for inclusion in the ypStage I group [22].

**Fig. 3:**
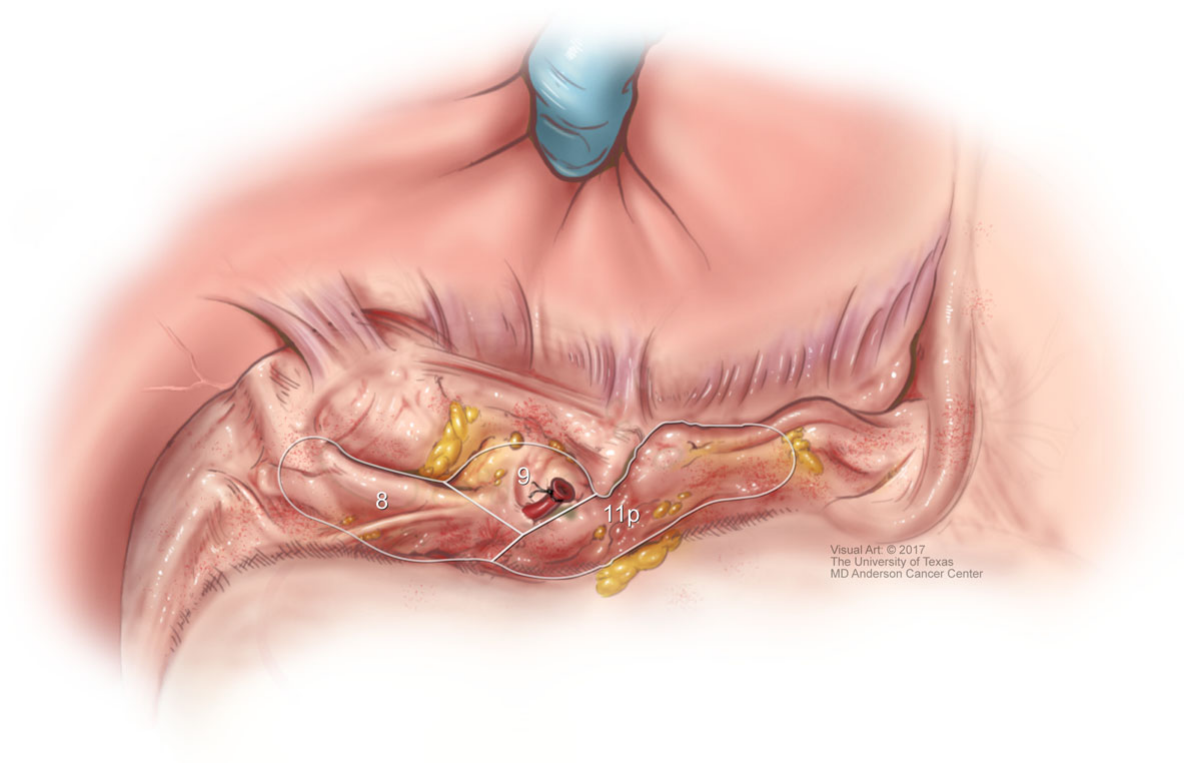
Extent of extended lymph node dissection for gastric cancer, labeled according to the Japanese classification system, including stations 8, 9, and 11p. (Reprinted from MD Anderson Cancer Center, with permission)

Extended lymph node dissection is routinely performed, as illustrated in Fig. 3, and only excluded for patients with T1a lesions or prohibitive comorbidities. Utilizing the Japanese classification of nodal stations, our extended lymph node dissection most often includes removing stations 8, 9, and 11p [23]. Eastern studies consistently demonstrate a benefit to extended lymph node dissection, while Western studies do not. The MRC trial and the Dutch trial failed to show a benefit with extended lymphadenectomy, primarily due to the increased complications attributed to pancreatectomy and splenectomy, although the Dutch trial showed an improvement in locoregional recurrence and disease-related survival for the D2 arm on long-term follow-up [24–27]. We acknowledge the lack of evidence, based on prospective randomized trials, and therefore routinely perform extended lymph node dissection, but only if it can be performed safely. The safety of D2 lymphadenectomy without routine pancreatectomy and splenectomy is supported by the Italian IGCSG-R01 trial, although benefit to extended dissection was only identified on subgroup analysis for patients with locally advanced gastric cancer and positive nodes [28, 29]. As an example of how these studies impact our current practice, if the extended lymph node dissection would result in blood transfusion, that otherwise would not be required, we would exclude the extended dissection. Based on the likely benefit in patients with advanced malignancy, impact of stage migration, low risk reported from expert centers, and single institution reports of an independent association with survival, numerous national gastric cancer guidelines recommend extended lymphadenectomy [5, 30–33].

## Outcomes

Increased postoperative morbidity and mortality after preoperative chemoradiotherapy is an important concern that has only recently been addressed in the setting of a randomized clinical trial for patients with resectable gastric cancer [17]. Preoperative chemotherapy and preoperative chemoradiotherapy for tumors localized to the esophagus or gastroesophageal junction have been demonstrated as safe treatment modalities with similar postoperative complication rates, death rates, and length of hospital stay based on Phase III trials [16]. In a retrospective review at MD Anderson of 200 patients undergoing upfront surgery, and 235 patients treated with preoperative chemoradiotherapy, we found similar postoperative morbidity and mortality rates [34]. The overall leak rate and symptomatic intra-abdominal fluid collection rate were 3.5 and 7.5%, respectively, and did not differ between treatment groups. Based on the level 1 evidence from other types of preoperative therapy, and our intra-institutional data, we consider preoperative chemoradiotherapy safe for patients with gastric cancer, although we continue to strive to improve tolerance and minimize complications in our frail patients [35, 36].

As a selective approach to patients with gastric cancer, and given the well-documented difficulty in administering postoperative therapy, patients treated with preoperative chemoradiotherapy should demonstrate improved survival compared to series of patients treated with upfront surgery. That appears to be the case based on retrospective data reporting overall survival in patients completing preoperative chemoradiotherapy and resection. In an MD Anderson series of 192 patients treated from 1995 to 2012, patients with AJCC pathologic stage 0, I, II, and III disease demonstrated 5-year overall survival rates of 69, 63, 56, and 38% [21]. On multivariate analysis, nodal status was the primary determinant of survival with 5-year overall survival rates of 67, 42, 43, and 0% for patients with AJCC N stage 0, 1, 2, and 3 disease, respectively [21].

The patterns of recurrence after treatment with preoperative therapy and gastrectomy can help identify future treatment modalities to improve survival. In a study of almost 500 patients who underwent margin negative resection, a total of 125 (26%) developed recurrence with the peritoneum as the most common organ of recurrence (49%), followed by the liver (21%) [37]. Recurrences were classified as locoregional in 15%, peritoneal in 49%, and nonperitoneal distant organ in 54% [37]. The use of prophylactic HIPEC is being investigated as a means to prevent peritoneal recurrence for locally advanced gastric cancer [38].

## Conclusion

In summary, the rationale for preoperative therapy is strong due to the difficulty in completing postoperative treatment. Patients with gastric and gastroesophageal adenocarcinoma demonstrate long-term survival on the order of 60% with induction chemotherapy and chemoradiotherapy. Trials in Western populations are needed comparing preoperative chemotherapy to chemoradiotherapy, which are currently ongoing and will inform the decision regarding the optimal treatment [17].
